# Microorganism community structure: A characterisation of agrosystems from Madeira Archipelago

**DOI:** 10.1111/1758-2229.13227

**Published:** 2024-01-24

**Authors:** Maria Cristina O. Oliveira, Carla Ragonezi, Sofia Valente, José G. R. de Freitas, Miguel A. A. Pinheiro de Carvalho

**Affiliations:** ^1^ ISOPlexis ‐ Centre of Sustainable Agriculture and Food Technology, Campus da Penteada, University of Madeira Funchal Portugal; ^2^ Centre for the Research and Technology of Agro‐Environmental and Biological Sciences (CITAB), Inov4Agro – Institute for Innovation, Capacity Building and Sustainability of Agri‐Food Production University of Trás‐os‐Montes and Alto Douro Vila Real Portugal; ^3^ Faculty of Life Sciences, Campus da Penteada University of Madeira Funchal Portugal

## Abstract

Microbial diversity profoundly influences soil ecosystem functions, making it vital to monitor community dynamics to comprehend its structure. Our study focused on six agrosystems in Madeira Archipelago, analysing bacteria, archaea, fungi and AMF through classical microbiology and molecular techniques. Despite distinct edaphoclimatic conditions and management practices, bacterial structures exhibited similarities, with Alphaproteobacteria at 18%–20%, Bacilli at 11%–18% and Clostridia at 9%–14%. The predominance of copiothrophic groups suggested that soil nutrient content was the driver of these communities. Regarding archaea, the communities changed among sites, and it was evident that agrosystems provided niches for methanogens. The Crenarchaeota varied between 15% and 29%, followed by two classes of Euryarchaeota, Methanomicrobia (17%–25%) and Methanococci (4%–32%). Fungal communities showed consistent composition at the class level but had differing diversity indices due to management practices and soil texture. Sordaryomycetes (21%–28%) and Agaricomycetes (15%–23%) were predominant. Conversely, AMF communities appeared to be also influenced by the agrosystem, with *Glomus* representing over 50% of the community in all agrosystems. These insights into microbial groups' susceptibilities to environmental conditions are crucial for maintaining healthy soil and predicting climate change effects on agrosystems' productivity, resilience and sustainability. Additionally, our findings enable the development of more robust prediction models for agricultural practices.

## Graphical abstract

Microbial diversity shapes soil ecosystems, necessitating monitoring for comprehension. Our study of Madeira's six agrosystems reveals consistent bacterial structures driven by copiotrophic groups, suggesting soil nutrient influence. Archaeal communities vary, providing niches for methanogens, while fungi show consistent class‐level composition influenced by management practices. Arbuscular mycorrhizal fungi are impacted by agrosystem dynamics. These insights are vital for soil health and predicting climate change effects, enhancing robust prediction models for sustainable agriculture.

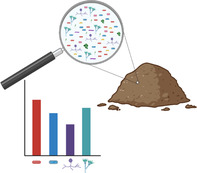

## INTRODUCTION

Soil quality has been defined as ‘the capacity of soil to function within ecosystem boundaries to sustain biological productivity, maintain environmental quality, and promote plant and animal health’ (Doran & Parkin, 1994). Agricultural soils are responsible for key ecological functions and services, including primary production of food, fibre, and fuel, nutrient cycling, carbon cycling, and storage, and water infiltration and purification (Schulte et al., 2014). Overall, agricultural soils contribute to the maintenance and productivity of agrosystems. However, the need to provide enough food for Earth's growing population has led to the pressure on biotic and abiotic resources of the agrosystems, resulting in unsustainable agriculture. Intensive management practices involving the use of synthetic fertilisers and pesticides, mechanical soil perturbation, and monocropping cause soil degradation, environmental pollution, and greenhouse gas emissions, which exacerbate climate change (Guzman et al., 2021; Hartmann & Six, 2023; Wang et al., 2020). Thus, the major challenge in this topic is how to balance the world's supply of food needs with the preservation of soil quality.

Soil biodiversity has been highlighted as a key component of healthy soil (Creamer et al., 2016; European Commission, 2020; Hermans et al., 2020), and microorganisms constitute a major portion of this biodiversity. They are considered drivers of ecosystem services, being responsible for many processes that are crucial for soil maintenance, and play key functions in numerous vital processes, such as carbon sequestration, and nutrient cycling, and can carry out important biochemical transformations (Hartmann & Six, 2023). They can also act through indirect mechanisms such as reducing toxicity by heavy metals and inducing resistance to pathogens.

Microorganisms are sensitive to environmental changes (Thomson et al., 2015; Zeilinger et al., 2016). Some studies demonstrate that microbial communities' composition, abundance, and/or diversity show a certain spatial distribution pattern with some environmental variables. Changes in those variables can affect directly or indirectly microbial communities' composition and dynamics (Liu et al., 2019). In the recent consensus statement ‘Scientists' Warning to Humanity: microorganisms and climate change’, scientists highlight the crucial role and global importance of microorganisms in mitigating climate change, stressing that the extent of the impact of climate change will heavily depend on microorganisms' responses, which are indispensable for reaching an environmentally sustainable future (Cavicchioli et al., 2019). Thus, it is imperative to understand the factors that shape community structure composition to fully comprehend the mechanisms impacting soil quality. Employing ecological methodologies, such as analysing the diversity and functionality of soil microbial communities, and conducting causal investigations of the effects of external factors on their structure, holds the potential to address those questions (Wang et al., 2020).

Knowledge about the microbial communities in agricultural soils is mostly derived from mainland agrosystems, which differ greatly from the conditions found in islands (Hartmann & Six, 2023; Li et al., 2020). Oceanic islands offer valuable opportunities for unravelling the interaction between evolutionary and ecological factors that drive community assembly and composition (Emerson & Gillespie, 2008; Whittaker et al., 2017) and are known to have peculiar characteristics that can influence the soil microorganisms (e.g. environmental conditions, the size of supported populations, species dispersal, soil parent materials, and soil formation (Li et al., 2020; Lin et al., 2019). Due to the size and orography of the territory, agriculture in Madeira is of the mountain type, showing a mixture of Subtropical and Mediterranean climatic elements. The landscapes, agrosystem structure and farm size contribute to a predominantly family‐based farming system, with low inputs, rotational practices, intercropping, or other poly‐cultural approaches. In this study, we aim to investigate the community structure of soil bacteria, archaea, fungi and arbuscular mycorrhizal fungi (AMF), in six locations, that differ in edaphoclimatic/atmospheric conditions and management practices, from the Madeira Archipelago. This work is part of an extensive collection of data, generated by the CASBio project: assessment and monitoring of agrobiodiversity and sustainability of agrosystems in the new climate scenarios (see Pinheiro de Carvalho et al., 2022). The project relies on the premise that climate change can be catastrophic to insular regions, such as the Madeira Archipelago, representing the loss of agricultural systems and the degradation of the land, focusing particularly on soil erosion and loss of productive potential (Lincoln Lenderking et al., 2021). In addition, for Madeira, Climate—Madeira Strategy (Gomes et al., 2015), based on Intergovernmental Panel on Climate Change models (Parry et al., 2007), estimates an anticipated increase in average temperature ranging from 1.4 to 3.7°C and a decrease in precipitation of 30% to 40% by the year 2070. Therefore, the goal of CASBio is to evaluate agrosystems sustainability in the expected climate scenarios for Madeira Archipelago and to develop knowledge and technology that can contribute to increasing the resilience of agriculture and promoting the local bioeconomy.

## EXPERIMENTAL PROCEDURES

### Agrosystems characterisation

The survey was conducted in the Madeira Archipelago located in the Atlantic Ocean, between the latitudes 33°10′–32°20′ N and longitudes 16°10′–17°20′ W, at 900 km south‐west from the mainland (Portugal) and 630 km west of the coast of North Africa (Madeira et al., 2007). Madeira Archipelago has two habited islands, Madeira and Porto Santo. Madeira Island is the largest and highest of the islands with a total area of 728 km^2^. Its soils are highly porous, dark‐coloured (andisols), developed from volcanic origins that typically occur in wooded highland areas (Ganança et al., 2007). Porto Santo is the other habited island with an area of 42.3 km^2^. This island's soil reflects the nature of the parent material and the dry climate, making the calcisols the major dominant soil group (Madeira et al., 2007). Due to the orography of the Madeira Archipelago and the size and structure of the agrosystems, family farming prevails and is mainly based on sustainable ancestral techniques, such as low input, rotation, intercropping, or pluricultural practices (Pinheiro de Carvalho et al., 2021, 2022).

**FIGURE 1:**
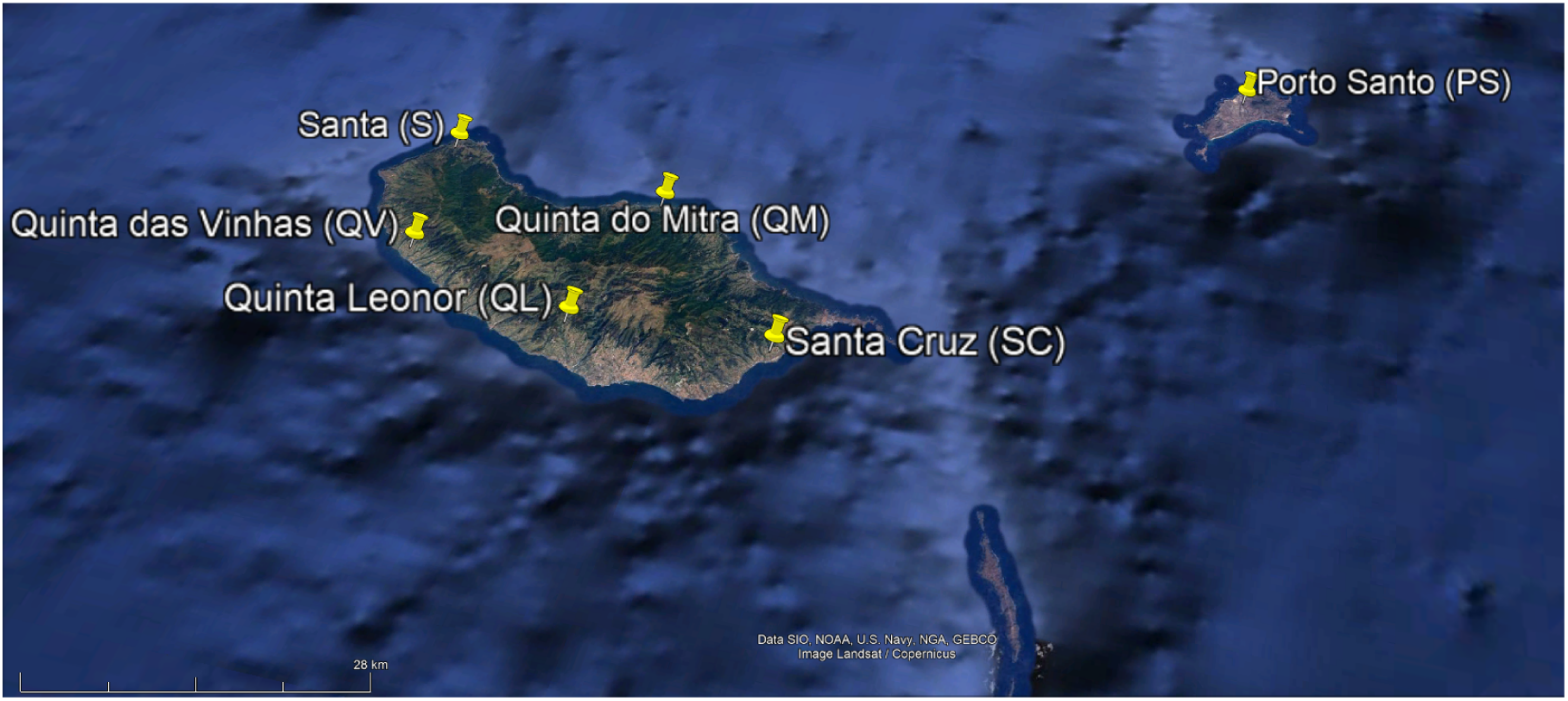
Sampled agrosystems from the Madeira Archipelago.

For this study, we analysed the soil's microorganisms of six agrosystems from the Madeira Archipelago (Figure 1). Important characteristics of the six studied agrosystems are detailed in Table 1. For the edaphic characterisation, the following parameters were used: pH, Organic Matter (OM), Assimilable Phosphorus (P), Assimilable Potassium (K), Cation Exchange Capacity (CEC), Nitrogen (Nitrate and Ammonium) and the Soil texture classification (Table 1). The climatic conditions of the agrosystems were evaluated with data obtained from 11 meteorological stations of the Portuguese Institute for Sea and Atmosphere, I.P. (IPMA, IP), and 6 meteorological stations (Davis Vantage Pro 2 Wireless) placed in situ or the vicinity. Climatic data were calculated from the daily data mean for the period under study. The climatic measures were: averages of the maximum and minimum temperature (°C), humidity (%), and accumulated precipitation (mm). For detailed methodology, see Ragonezi et al. (1902).

**TABLE 1:** Important characteristics of the six studied agrosystems.

Characteristics	Quinta das Vinhas (QV)	Quinta Leonor (QL)	Quinta do Mitra (QM)	Santa (S)	Santa Cruz (SC)	Porto Santo (PS)
Coordinates	32°44′4.214″ N, 17°11′13.564″ W	32°41′ 17.228″ N, 16°59′40.740″ W	32°49′46.085″ N, 16°56′50.255″ W	32°49′46.085″ N, 16°56′50.255″ W	32°41′54.460″ N, 16°47′0.575″ W	33°4′38.676″ N, 16°20′50.975″ W
Altitude/m	331	729	58	549	183	84
Average temperature (°C)^a^	18.5 ± 1.61	17.3 ± 1.37	17.6 ± 1.31	17.0 ± 1.80	19.9 ± 1.30	19.1 ± 1.35
Average maximum temperature (°C)^a^	25.3 ± 1.01	24.9 ± 1.77	24.05 ± 1.60	24.4 ± 1.33	24.5 ± 1.76	23.8 ± 1.40
Average minimum temperature (°C)^a^	13.7 ± 1.52	11.2 ± 1.32	12.85 ± 1.35	11.6 ± 2.47	15.7 ± 1.52	13.7 ± 1.65
Mean humidity (%)^a^	64.8 ± 2.85	65.9 ± 5.32	77.23 ± 5.04	77.4 ± 2.93	67.6 ± 0.88	74.6 ± 1.58
Accumulated precipitation (mm)^a^	127.1 ± 60.5	119.9 ± 56.47	216.74 ± 90.23	142 ± 76.03	122.3 ± 79.96	102.6 ± 39.03
Cultivated crop/Cropping system	Vineyard monoculture	Apple monoculture	Vegetables	Vegetables	Banana monoculture	Vineyard monoculture
Mode of production	Transitioning to organic farming	Organic farming	Organic farming	Organic farming	Traditional	Traditional
pH [KCl]	5 ± 1.10	5 ± 0.08	6 ± 0.06	5.2 ± 0.05	4.4 ± 0.08	7.3 ± 0.12
OM (%)	3.1 ± 0.21	6.7 ± 0.76	4.2 ± 0.40	8.8 ± 0.93	5.7 ± 0.41	1.9 ± 0.28
Assimilable P [ppm]	134.1 ± 4.72	114.5 ± 167.88	801.7 ± 93.56	194.5 ± 62.36	557.3 ± 81.74	923.5 ± 90.26
Assimilable K [ppm]	405.5 ± 86.13	540 ± 89.15	570 ± 98.68	870 ± 184.90	982 ± 261.15	1420 ± 510.29
CEC [meq/100 g]	36.5 ± 3.92	55.4 ± 7.25	68 ± 6.67	47.1 ± 6.65	60.7 ± 8.01	30.1 ± 3.29
Nitrogen (Nitrate) [ppm]	22.2 ± 7.17	61 ± 23.49	68.3 ± 18.38	73.8 ± 21.23	98.8 ± 28.19	23.4 ± 5.82
Nitrogen (Ammonium) [ppm]	3.9 ± 0.93	4.9 ± 1.33	4.5 ± 1.36	4 ± 1.75	4.7 ± 2.55	2.7 ± 0.22
Soil texture classification^b^	Silt loam	Silt clay	Silt loam	Silt loam	Silt clay loam	Silt clay

*Note*: ± denotes the standard error of the mean.

^a^ Two‐year average.

^b^ Texture triangle according to USDA (Soil Science Division Staff, 2017).

### Sampling and microbiologic parameters

The analysis of soil microbiota followed a classical and molecular approach. Soil samples were collected according to Paetz and Wilke (2005), with modifications. Briefly, soil sub‐samples were collected in a zigzag pattern along the sampling sites, at a depth range of 15 to 20 cm. Sub‐samples were mixed to create a representative sample of the local soil. Soil samplings were collected every 3 months, for 2 years (eight missions in total), to cover the four seasons and possible microbial fluctuations over the year.

For the classical microbiology approach, nitrogen‐fixing and denitrifying bacteria, total bacteria and total fungi were quantified in triplicates, following the dilution method and inoculation in proper media, mannitol agar (MA), nitrate broth (NB), potato dextrose agar (PDA), and nutrient agar (NA). Results were expressed in colony‐forming units (CFU) or most probable number (MPN) per gram of dry soil.

According to Yeates et al. (1998), molecular analysis was performed with the extraction of total genomic DNA from soil samples, in sextuplicate. Bacteria, fungi, archaea and AMF were studied using the Terminal Restriction Fragment Length Polymorphism (T‐RFLP) approach. The amplification of fragments by polymerase chain reaction (PCR) was performed with the labelled forward primer (5′ 6‐FAM). For a detailed primer sequence, see Pinheiro de Carvalho et al. (2022). The amplified fragments were cut by two restriction enzymes for each group of microorganisms: Hinf I/Hae III for bacteria and fungi, Hae III/Alu I for Archaea and Hinf I/Mbo I for AMF. The forward fragments that resulted from the enzymatic cutting, termed as terminal restriction fragments (T‐RFs) were analysed by capillary electrophoresis and determined by Genescan analysis software (Applied Biosystems‐Stabvida, Portugal). Then, the software T‐REX (Culman et al., 2009) was used to process and obtain the matrices. The web‐based tool MiCA was used to infer the community composition of the microorganisms' groups under study (Shyu et al., 2007).

### Data analyses

Alpha‐diversity was measured, using the following indexes: Species Richness (R), Shannon–Wiener Diversity (H′) and Corrected Evenness (E′), calculated with the statistical programme Community Ecology Parameter Calculator (ComEcoPaC) version 1.0 (Drozd, 2010), according to Nóbrega et al. (2021). Comparisons among groups were performed using the statistical package for the social sciences (SPSS, version 23.0, IBM Corp, Armonk, NY, USA). Data were tested for normality (Shapiro–Wilk), then submitted to One‐way ANOVA and Tukey HSD test, expressing differences at *p* ≤ 0.05. When normality was not observed, we followed Templeton (2011) to normalise the data. CFU, MPN quantification and alpha‐diversity plots were generated using the function boxplot from package graphics and theme update from package ggplot2 (Wickham, 2016) in RStudio Version 1.3.1056. Beta‐diversity was determined with the average abundance of T‐RFs found in each agrosystem, using the function vegdist from package vegan (Oksanen et al., 2022), method ‘bray’ (for Bray‐Curtis index [Bray & Curtis, 1957]) and plotted using the function hclust from package stats, method ‘average’ (for UPGMA clustering). Graphics for taxonomic composition were generated in Excel (Microsoft Office 2019) and show the average percentage of each class or genus found in all samplings that were determined for the two selected restriction enzymes for each microbial group. Venn diagrams and the combined UpSet plots were generated using the tool Venny 2.1 (Oliveros, n.d.) and UpSetR (Conway et al., 2017) package in R. These diagrams and plots show the percentage and number, respectively, of shared T‐RFs generated by the two enzymes used in each microorganism group, among agrosystems, management practices and soil texture.

## RESULTS AND DISCUSSION

### Quantification of bacteria and fungi

**FIGURE 2:**
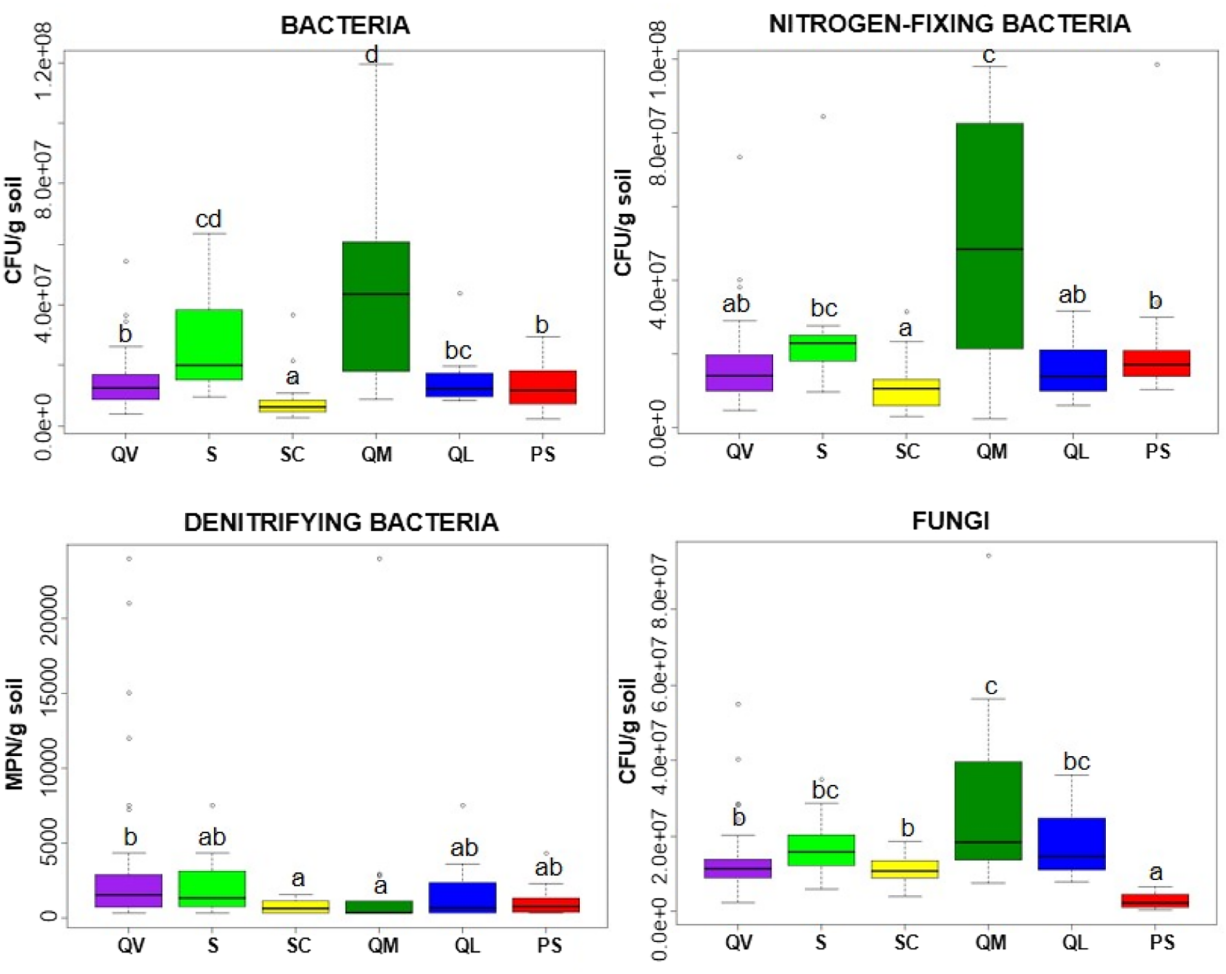
Variation in total bacteria, fungi, nitrogen‐fixing, and denitrifying bacteria abundance for the 2 years. Boxes with different letters indicate a significant difference among them (ANOVA, *p* ≤ 0.05). The letter ‘a’ corresponds to the lowest values.

Regarding the quantification of the microorganisms, nitrogen‐fixing bacteria, total bacteria, and fungi showed values between 10^6^ and 10^7^ CFU/g in dry soil. Denitrifying bacteria showed values around 10^3^ MPN/g in dry soil (Figure 2).

ANOVA analysis showed significant differences (*p* ≤ 0.05) among the groups of microorganisms in the different agrosystems. The boxplots in Figure 2 show the variation of the studied microorganism groups, expressed in CFU/gram of dry soil, found in the samples from the agrosystems. Quinta do Mitra (QM) had the highest values for Bacteria, N‐fixing bacteria and Fungi. Santa (S) also had higher bacteria values than the other four sites. Concerning the denitrifying bacteria, Santa Cruz (SC), and QM, showed lower values and less variation among samplings. Porto Santo (PS) was the site with smaller values for fungi.

The values, in CFU/g of soil, obtained for bacteria and fungi are similar to the average values detected in other soil samples from agricultural systems in Madeira Archipelago (ISOPlexis Germplasm Resources Information Network, 2023) and in agreement with the expected values for bacteria (10^6^ to 10^9^ CFU/g soil) (Whitman et al., 1998) and for fungi (10^3^ to 10^6^ CFU/g soil) (Lawlor et al., 2000; Molina‐Guzmán et al., 2018).

Statistical analysis showed that microbial abundance changed depending on the agrosystem. The highest abundance was observed for QM in three groups (Figure 2). QM production is centred around a mixture of vegetables produced via organic farming. Compared with the other agrosystems, QM has a median amount of OM and N (nitrate and ammonium) but the highest CEC, and the soil was classified as Silt Loam (Table 1). Soil texture can have a big influence on the abundance of microorganisms, because the structure, such as aggregation and pore connectivity, regulates the flow of water, oxygen and nutrients throughout the system. For example, in Chodak and Niklińska (2010), soil texture was more important to the microbial properties of mine soils than the vegetation composition. Hamarashid et al. (2010) evaluated the effects of soil texture on the abundance of bacteria and fungi in distinct soils from the Sulaimani governorate (Iraq). Differently from our results, the highest value for bacterial populations was detected in Silty Clay Loam and Clay Loam soils. For fungi, the authors did not observe any differences among soils' textures, but in our study, QM registered the highest value, and PS, a vineyard monoculture with a Silt Clay texture, had the lowest fungi abundance. Heritage et al. (2003) stated that sandy soils cannot properly retain water, draining it very fast. On the other hand, clay loam soils retain water and hold nutrients for a longer period, which could explain the high levels of bacteria in soils with clay contents observed by Carney and Matson (2005). In our study, although QM did not present a high content of clay (20%), it was the agrosystem with higher bacteria abundance. It is a fact that other characteristics of the area influenced the results such as the CEC values (related to soil nutrient availability), the cropping system, and land use.

### Composition of microbial communities

The T‐RFLP method and the chosen enzymes for each group of microorganisms allowed the detection of 142 different T‐RFs for Bacteria, 124 for Archaea, 178 for fungi and 189 for the AMF group. According to the comparison between T‐RFLP profiles obtained and public databases, using the MiCA tool, the T‐RFs belong to 17 classes of Bacteria, 8 of Archaea, 11 of fungi and 4 genera of AMF. It was not possible to classify at the class level an average of 28% of the detected T‐RFs for Bacteria, 71% for Archaea and 16% for Fungi. Regarding AMF, there was an average of 77% of the detected T‐RFs with no correspondence at a genus level.

**FIGURE 3:**
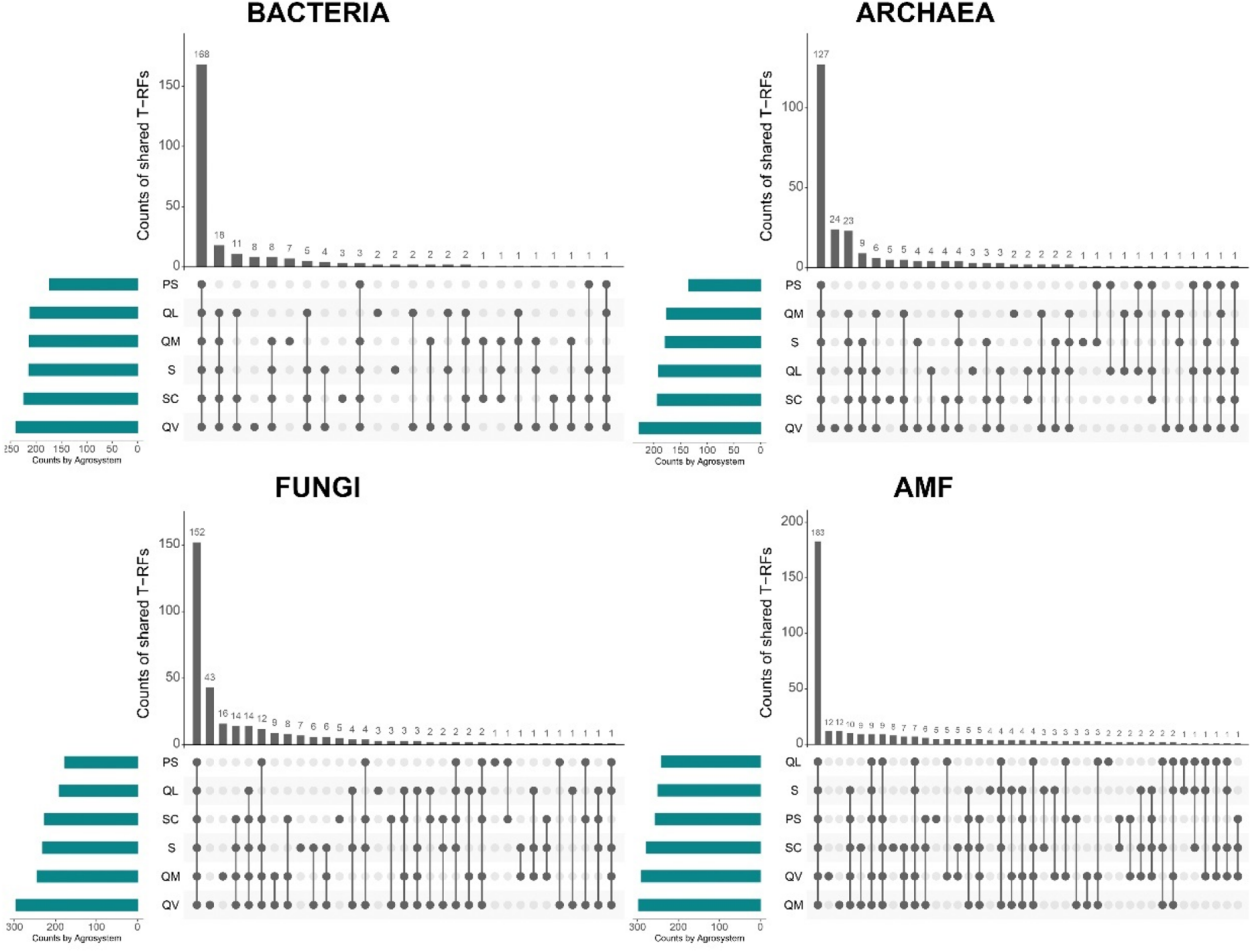
UpSet plot showing how many unique T‐RFs of bacteria, archaea, fungi and AMF are shared between and among agrosystems.

The UpSet plots of the microbial communities showed that the biggest part of the detected T‐RFs is shared by the six agrosystems (Figure 3). These T‐RFs are the resident microbiota of the archipelago of Madeira, due to their persistent presence in all agrosystems, despite the distinct geographic location and edaphoclimatic conditions. The agrosystem PS, that is located on a different island than the others, did not showed any unique T‐RF for bacteria and archaea. However, the other five agrosystems showed unique T‐RFs for all groups, especially in fungi and AMF communities, indicating that the conditions found in the agrosystems that they are present are more favourable to their settlement.

#### Taxonomic composition of prokaryotic communities

**FIGURE 4:**
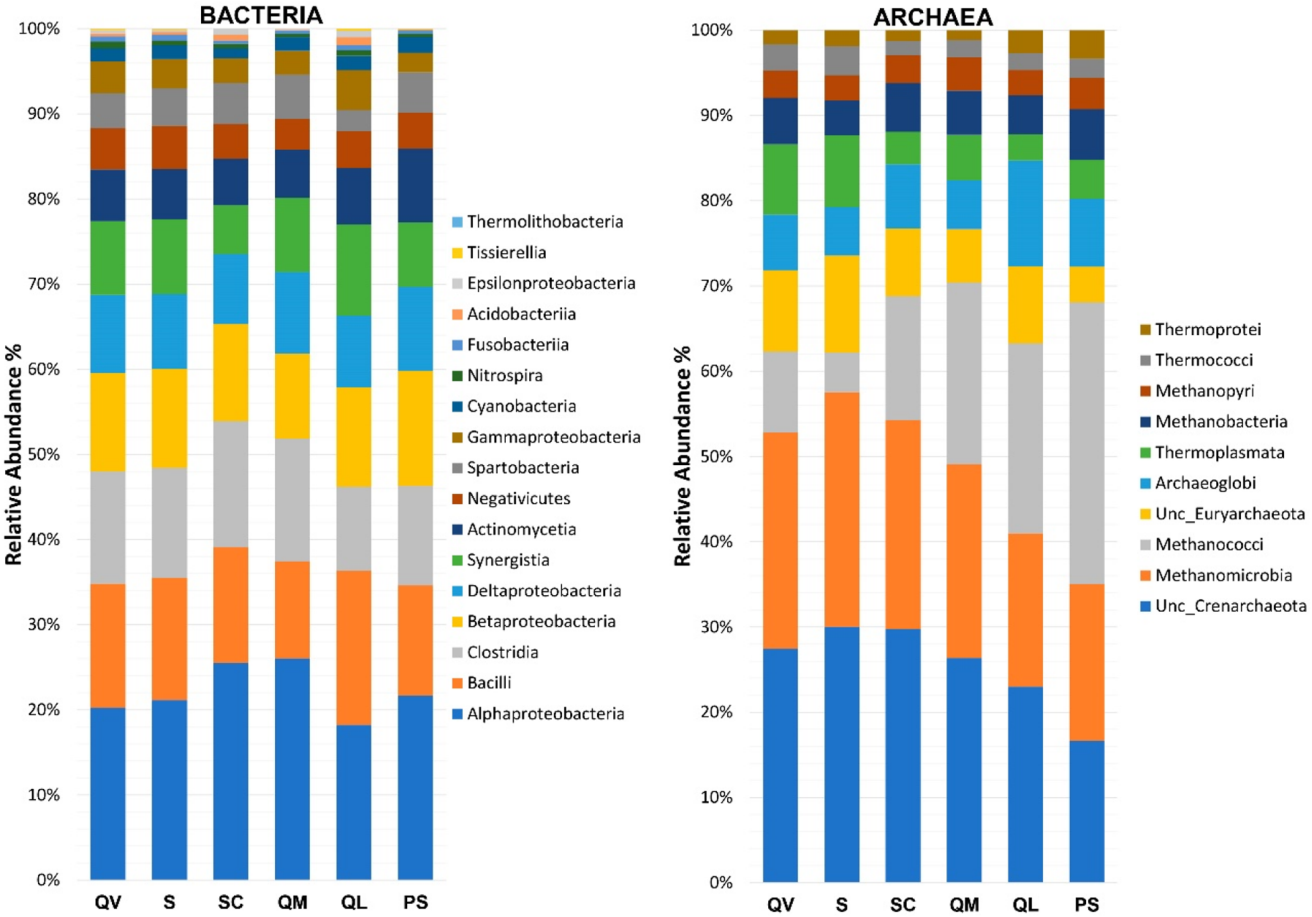
Relative abundance (%) of T‐RFs, identified at a class level, for Bacteria (left) and Archaea (right) communities.

The generated profiles of Bacteria found for the six agroecosystems at the class level were very similar, suggesting that the different environmental conditions and general management practices found in those agrosystems had little impact on communities' composition, but the community composition profiles of Archaea were less homogeneous among sites (Figure 4).

The most predominant classes found for Bacteria in all agrosystems were Alphaproteobacteria (phylum Proteobacteria), followed by Bacilli and Clostridia (phylum Firmicutes), all known to be copiotrophic groups, that is, associated with nutrient‐rich environments (Wongkiew et al., 2022) (Figure 4, left). The class of Alphaproteobacteria, in these samples, was mainly composed of members of the genera *Rhizobium* and *Mesorhizobium*, both with the ability to penetrate the roots of plant legume species, producing root nodules in which N fixation occurs. The ammonia produced in this process is then assimilated by the plants (Weir et al., 2004). The molecular results agreed with the quantification results, which showed high quantities of CFU/g of soil for the Nitrogen‐fixing bacteria group in all agrosystems (Figure 2). The predominance of Clostridia in these soils is also important, as the members of the genus *Clostridium* are known to be capable to ferment several carbohydrates, contributing to organic matter decomposition in anaerobic conditions (Kim, Samaddar, et al., 2021; Tanahashi et al., 2005).

The class Acidobacteriia is ubiquitous and among the most abundant bacteria in soil, but in this study, it was found in low relative abundance in all agrosystems. Some studies showed a negative impact of agriculture on the relative abundance of the phylum Acidobacteria. A meta‐analysis revealed that agricultural soils have a lower relative abundance of Acidobacteria than natural soils, in arid, continental and temperate regions (Trivedi et al., 2016). Members of this phylum are classified mainly as oligotrophs (Kalam et al., 2020), so the results observed in this study may be related to the contents of nutrients in these soils, derived from agricultural practices. Oligotrophs exhibit slow growth, low metabolic rates and usually low population density, and have high affinity to substrates with low nutrient provisions (Hartmann & Six, 2023).

Kim, Lee, et al. (2021) proposed the phyla Proteobacteria and Acidobacteria as bioindicators for land‐use change since they tend to increase and decrease, respectively, with agricultural management practices that lead to changes in soil physicochemical parameters, mainly the increase in the concentration of nutrients.

Archaea showed some differences among sites (Figure 4, right). While the class Methanomicrobia was found to be more predominant in QV, S and SC, the other three sites had a higher relative abundance of Methanococci. Both belong to the phylum Euryarchaeota and are predominantly methanogens (Bapteste et al., 2005). Methanogens play a central role in the global carbon cycle, as they carry out the last stages of the degradation of organic material in anaerobic conditions. Much of the methane produced in the process is then converted into carbon dioxide by methanotrophs, mainly bacteria. However, some are released into the atmosphere, contributing to the greenhouse effect (Anderson et al., 2009; Buan, 2018). Methanosarcinales was the predominant order, corresponding to 30%–44% of the class Methanomicrobia found in all agrosystems. Many members of Methanosarcinales are capable of more than one methanogenesis pathway, while the remaining methanogens, as far as the scientific community knows, are mainly hydrogenotrophic, which is also the case of Methanococci, or in a few cases methylotrophic (Buan, 2018). The hydrogenotrophic pathway leads to the production of CH_4_ and H_2_O from CO_2_ and molecular hydrogen (H_2_), whose presence largely determines the occurrence of this process (Li et al., 2021; Shima et al., 2020). So, probably this was the reason why Methanococci was well represented in some agrosystems but not in others, giving space to more flexible methanogenic archaea to grow, such as Methanosarcinales.

Although the phylum Crenarchaeota was well represented, Euryarchaetoa had the highest relative abundance in all the sites. Interestingly, Euryarchaeota is not the dominant phylum found for most soils in literature. Instead, the phyla Thaumarchaeota and Crenarchaeota are predominant (Karimi et al., 2018; Kemnitz et al., 2007; Megyes et al., 2021; Taffner et al., 2020; Truu et al., 2020). Euryarchaeota, where methanogens are included, was the dominant phylum mainly in rice paddy soils, due to the anoxic environment created by the flooded fields (Conrad et al., 2008; Hester et al., 2022). However, some studies showed that methanogens are ubiquitous also in aerated soils (Angel et al., 2012; Hofmann et al., 2016; Wang et al., 2015).

#### Taxonomic composition of eukaryotic communities

**FIGURE 5:**
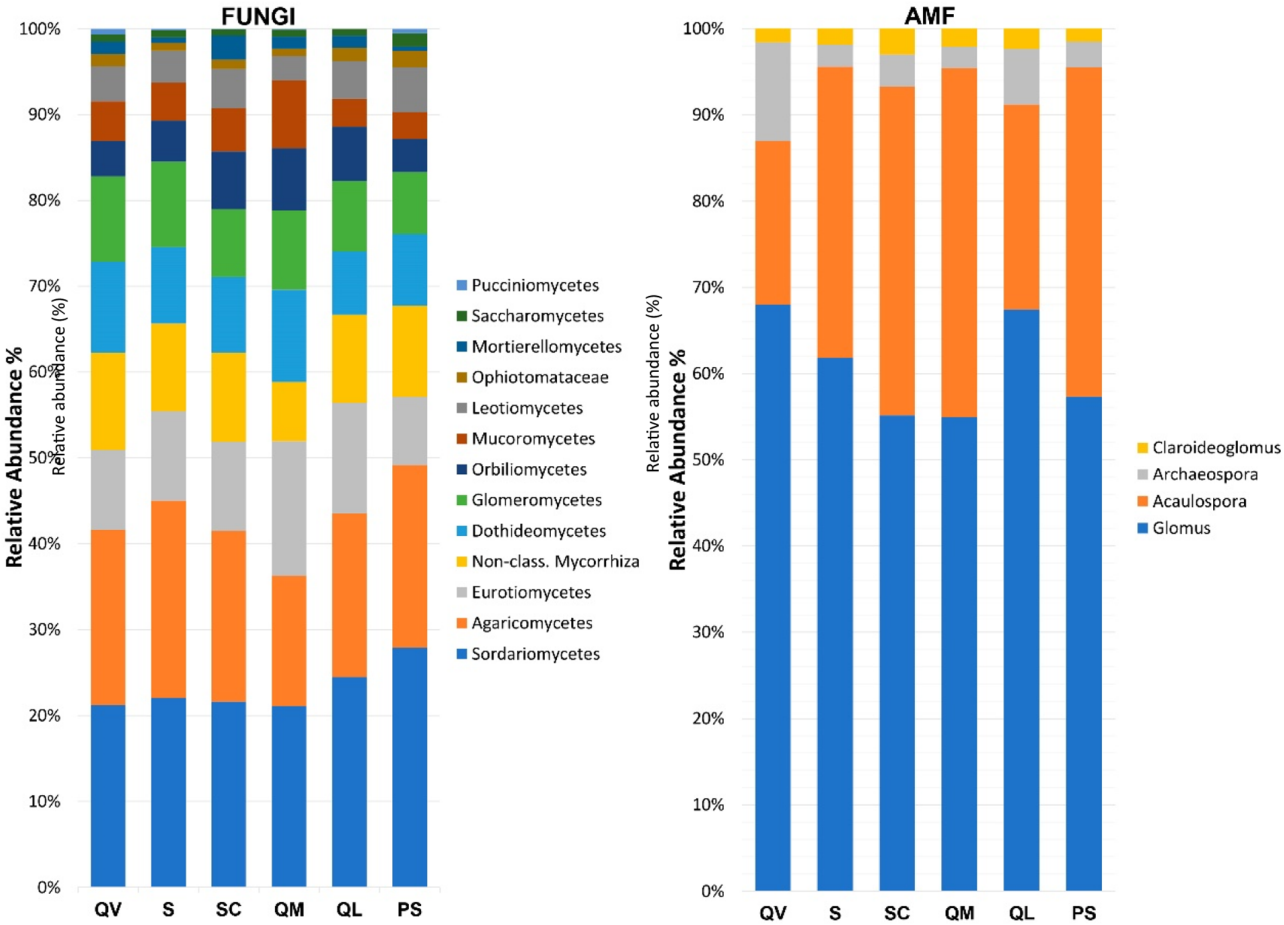
Relative abundance (%) of T‐RFs, identified at the class level, observed for Fungal (left) communities, and T‐RFs, identified at a genus level, observed for AMF (right) communities.

As with Bacteria, the profiles of Fungi found for the six agrosystems at the class level were very similar, and as with Archaea, the community composition profiles of the AMF group were less homogeneous among sites (Figure 5).

Sordariomycetes and Agaricomycetes were the fungal dominant classes in all agrosystems (Figure 5, left). Members of Sordariomycetes are abundant in the soil as pathogens, endophytes, mycoparasites and saprobes (Nguyen et al., 2016; Zhang et al., 2006). Koechli and colleagues identified Sordariomycetes as the most abundant decomposers of cellulose‐rich organic materials (Koechli et al., 2019). Another study also showed that saprotrophic Sordariomycetes were enhanced with the addition of sawdust to the soil (Clocchiatti et al., 2021). The most predominant genera found in these agrosystems were related to *Fusarium*, *Trichoderma, Acrostalagmus*, *Metarhizium*, *Daldinia* and *Chaetomium*. According to the literature, these genera are associated with organic matter decomposition and plant protection (Francis, 2019; Rubini et al., 2005; Soytong et al., 2021; Zin & Badaluddin, 2020). However, *Fusarium* species, in agriculture, are also widely associated with plant pathogenicity and can cause serious yield losses (Arie, 2019; Blanco & Aveling, 2018).

Agaricomycetes are the most abundant class of fungal communities from forest ecosystems, carrying important roles, such as wood‐decayers and ectomycorrhizal symbionts (Dang et al., 2018; Wu et al., 2019; Zhu et al., 2023). In this study, it was the second most abundant class and was well represented in all the agrosystems (12.3%–19.4% of all T‐RFs). Most of the studies analysing fungal communities in agricultural soils found Sordariomycetes as the dominant class, followed by other classes of Ascomycota, like Dothideomycetes and Eurotiomycetes. Agaricomycetes often account for much less than 10% of relative abundance (Balami et al., 2021; Clocchiatti et al., 2021; Dang et al., 2018; Fraç et al., 2018; Klaubauf et al., 2010; Naumova et al., 2022). The soil amendment with wood‐based materials, such as beech, seems to lead to an increase in Agaricomycetes (Clocchiatti et al., 2023; Malewski et al., 2023). Possibly, the application of organic compost commonly used in Madeira's agrosystems increases the concentration of materials in soil for which Agaricomycetes have an affinity.

The class Glomeromycetes and other non‐classified mycorrhiza were well represented in all agrosystems. *Glomus* was the dominant genus, followed by *Acaulospora* (Figure 5, right). *Glomus* accounted for more than 50% of the identified T‐RFs at a genus level, in the six sites, while *Acaulospora* ranged from 19.0% in QV to 40.4% in QM. *Glomus* has been reported as a common and dominant genus found in different land use systems and altitudes (Flores et al., 2022; Guzman et al., 2021; Zhang et al., 2021). Our results support the studies that observed the occurrence of *Glomus* in a wide range of environmental conditions and ecological niches, showing the adaptability of this genus (Fall et al., 2022; Zhang et al., 2021).

*Acaulospora* is also well‐represented in most soils and in some cases is the dominant genus. For example, a study carried out in the Azores Archipelago showed that Acaulosporaceae are the dominant members of native forests, and this result was attributed to the probable effect of the high content of OM, soil available N, and low pH (Melo et al., 2020). This can explain the observed relative abundance in agrosystems from Madeira Island (Table 1). And it could also be the reason why QV showed a lower relative abundance of *Acaulospora*, compared to the other agrosystems. However, in the case of QL, it was probably due to the culture (apple trees) and other spontaneous species occurrences (Davison et al., 2011; van Geel et al., 2015). Interestingly, the agrosystem from Porto Santo Island (PS) had a completely different soil profile regarding OM, pH and available N, but still showed a similar pattern of relative abundance to SC and QM.

*Glomus* (Deveautour et al., 2018) and *Archaeospora* (Kozjek et al., 2021) are associated with drought stress. In fact, QV is experiencing a decrease in precipitation over the years, with a soil water deficit between April and October (Pinheiro de Carvalho et al., 2022). This explains the higher relative abundance of those two genera in this agrosystem.

### Alpha and beta‐diversity

**FIGURE 6:**
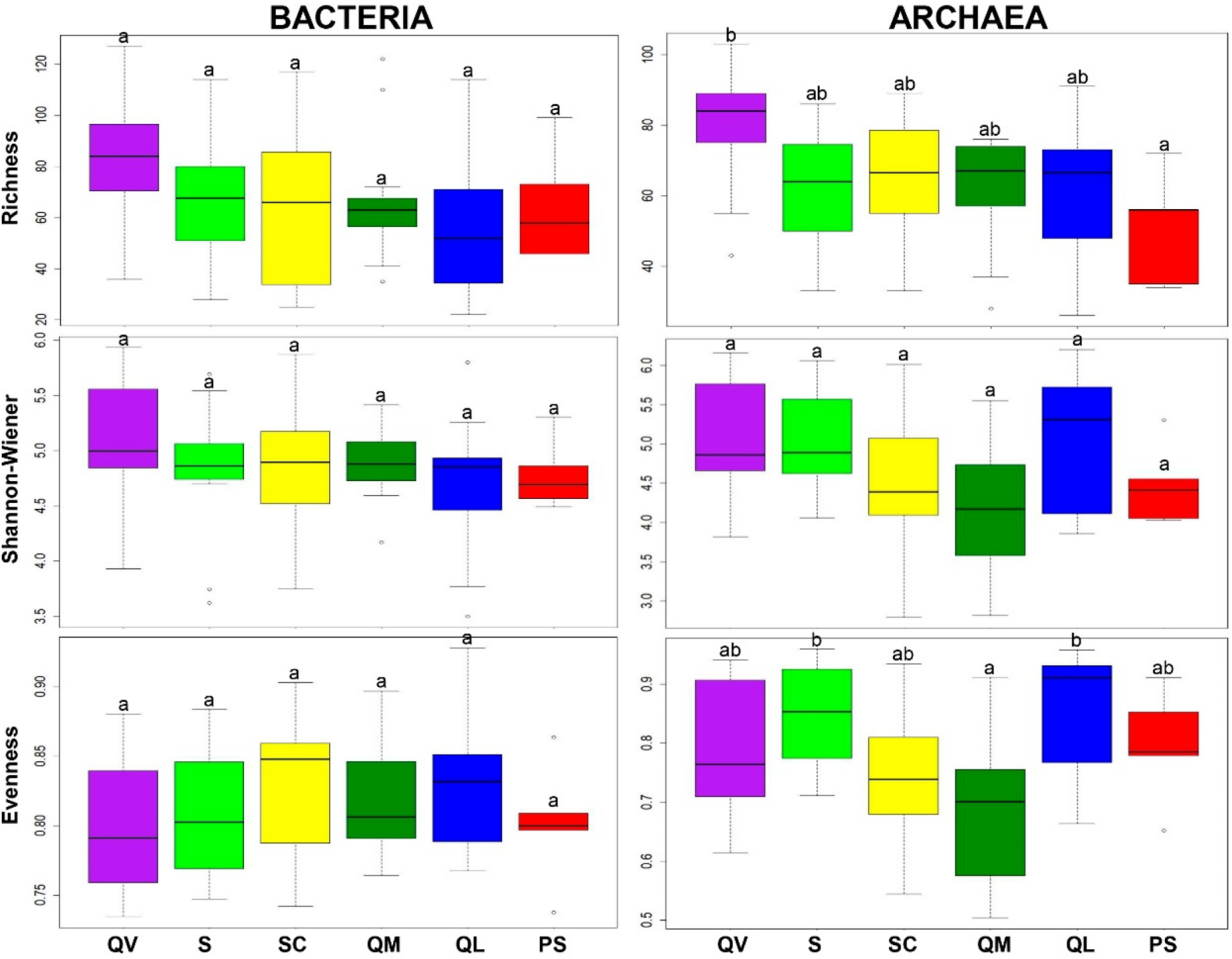
Variation in the T‐RF Richness (R), Shannon–Winner diversity (H′) and Corrected Evenness (E′) of Bacteria and Archaea by agrosystem. Different letters above the boxes indicate a significant difference between them (ANOVA, *p* ≤ 0.05). The letter ‘a’ refers to the lowest values.

It is possible to observe that in all agrosystems there was variability for the three calculated indexes, that were used for the alpha‐diversity analysis, T‐RF Richness (R), Shannon–Wiener (H′), and Corrected Evenness (E′). For Bacteria, there were no significant differences (ANOVA, *p* ≥ 0.05) observed among agrosystems for the three diversity indexes (Figure 6, left). Even with no statistical difference, QV is the agrosystem with the highest R in a sample, QV and SC for H′ and QL for E′.

For Archaea, significant differences (ANOVA, *p* ≤ 0.05) were observed among agrosystems for the two diversity indexes, with the exception of H′ (Figure 6, right). Similarly to Bacteria, regarding the richness of Archaea, QV was the agrosystem with the highest values and had significant differences when compared to PS (ANOVA, *p* ≤ 0.05). Regarding the evenness, statistical differences (ANOVA, *p* ≤ 0.05) were observed in QM (with the lowest values) and S and QL (with the highest values).

**FIGURE 7:**
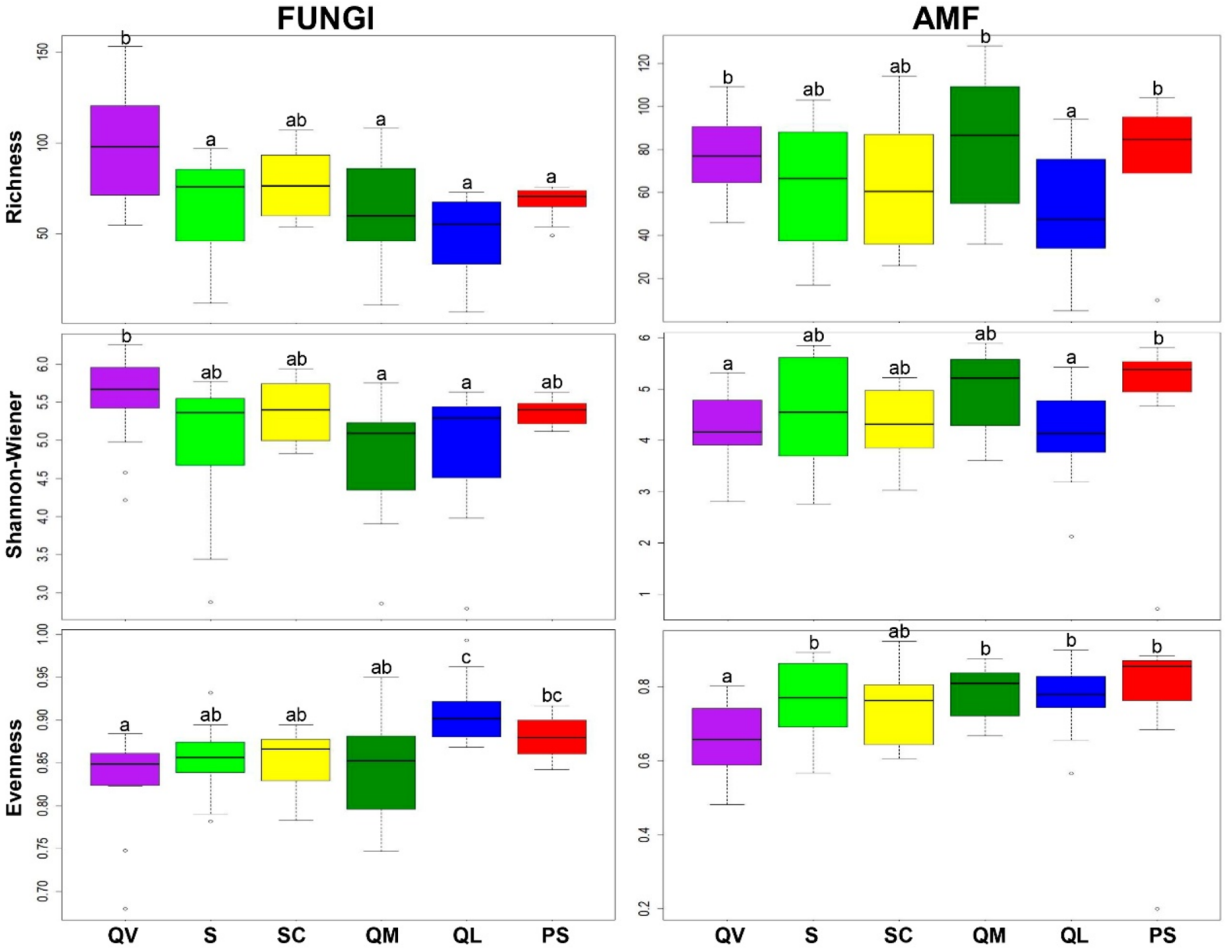
Variation in the Species Richness (R), Shannon–Winner diversity (H′), and Corrected Evenness (E′), of Fungi and AMF by agrosystem. Different letters above the boxes indicate a significant difference between them (ANOVA, *p* ≤ 0.05). The letter ‘a’ refers to the lowest values.

For fungi (Figure 7, left), QV continued to be the agrosystem with the highest and significantly different values (ANOVA, *p* ≤ 0.05), for R (except SC) and H′ (except S, SC and PS), but not for E′, which was QL. Regarding AMF (Figure 7, right), the highest and significantly different values of R were for QV, QM and PS, with being QL the lowest. PS also presented the highest value for H′. QV had the lowest value regarding E′.

**FIGURE 8:**
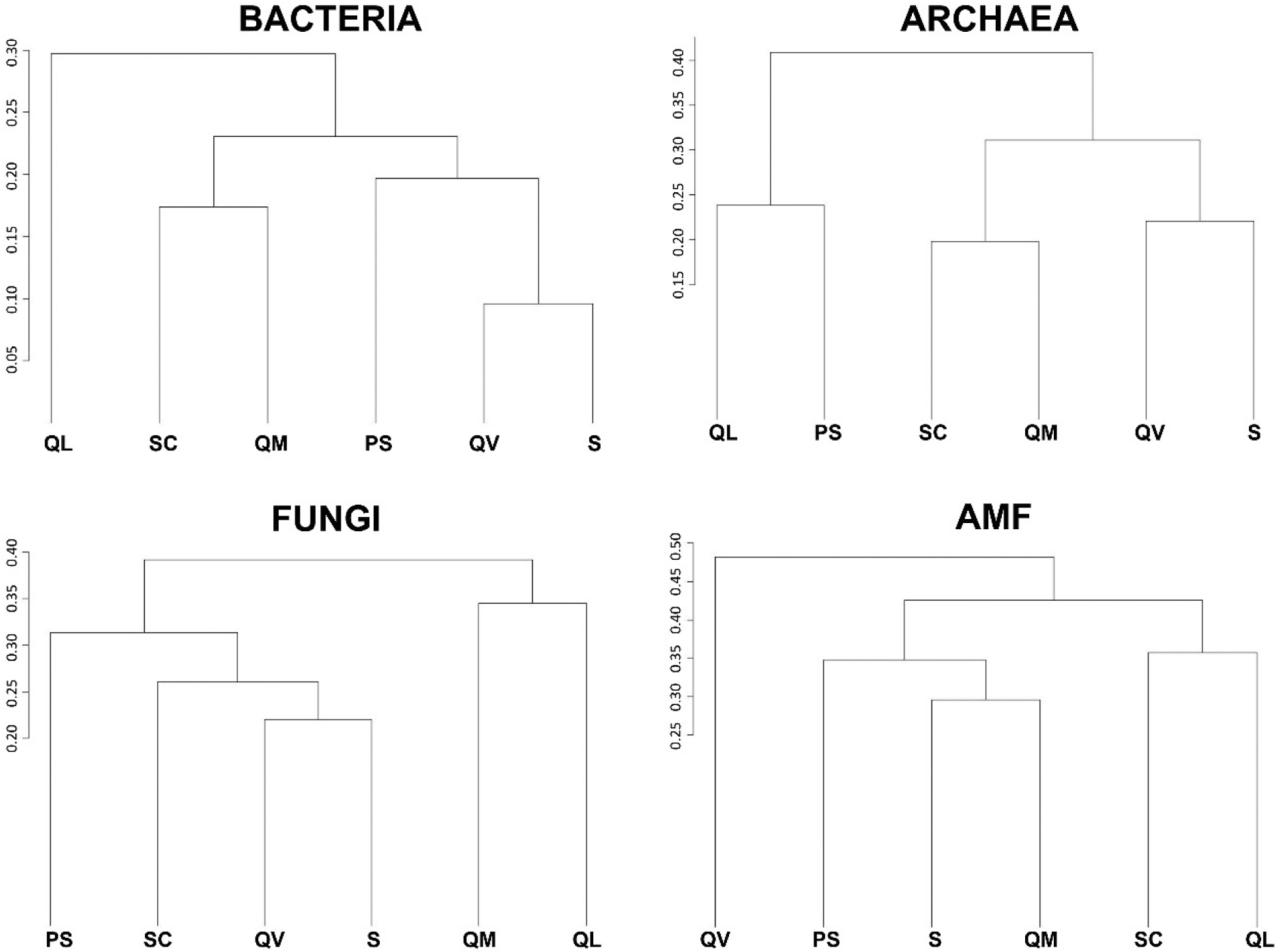
Dendrogram of the beta‐diversity (compositional dissimilarity between sites).

For the beta‐diversity assessment, the Bray–Curtis dissimilarity index was used, which ranged between 0 and 1, where 0 is for two sites with matching species composition, and 1 is for two sites that do not share any species. In our study, the dendrogram generated with the index showed that, in all studied areas and all microbial groups, the value was below 0.5 (Figure 8). AMF was the group that presented the highest value of dissimilarity and Bacteria the lowest. QL was the agrosystem that presented the most dissimilarity in the Bacteria group and QV in the AMF group.

In ecology, alpha‐diversity (taxa within a single sample) and beta‐diversity (spatial variation among samples) have been widely applied to evaluate microbial community variations (Guo et al., 2022). In our study, although Bacteria alpha‐diversity was the only one that did not differ significantly among sites, overall the communities did not present great variation. The beta‐diversity followed the same trend, being Bacteria the group with the smallest values, and AMF the highest, indicating their higher dispersion, but neither passed the threshold of 0.5. Flores‐Rentería et al. (2016), evaluated how the influence of the agricultural matrix, affects the composition and the functioning of soil microbial communities in holm oak forests from the Mediterranean territory and observed that bacterial and fungal richness did not vary along fragments, nor the Shannon and Evenness indexes. Beta‐diversity varied from 0.2 to 0.5 across samplings at three agricultural matrix influence levels, under a canopy or in open areas, in fragmented holm oak forests. This follows the value observed by Walters and Martiny (2020) where the average beta‐diversity of agriculture soils was around 0.5 (Flores‐Rentería et al., 2016). Jiao et al. (2018) in fields reforested from agricultural usage, observed that the fungal communities showed the highest beta‐diversity followed by Archaea and Bacteria. Although shifts in alpha‐diversity may occur while beta‐diversity remains stable, or vice versa (Gossner et al., 2013), this was not the case in this study. Anyhow, an integrated analysis of both indexes is critical when assessing changes over time across many microbial communities.

### Effect of different management practices and soil texture on microbial communities

The Venn diagrams were constructed to analyse the percentage of shared T‐RFs among different management practices and soil textures, and were complemented with UpSet plots (Figures S1 and S2). Agrosystems were grouped according to their cultivated crops (vegetables, banana, vine, apple trees), cropping system (monoculture or polyculture), mode of production (traditional, organic farming and transition to organic farming) and soil texture (silt loam, silt clay loam and silt clay).

**FIGURE 9:**
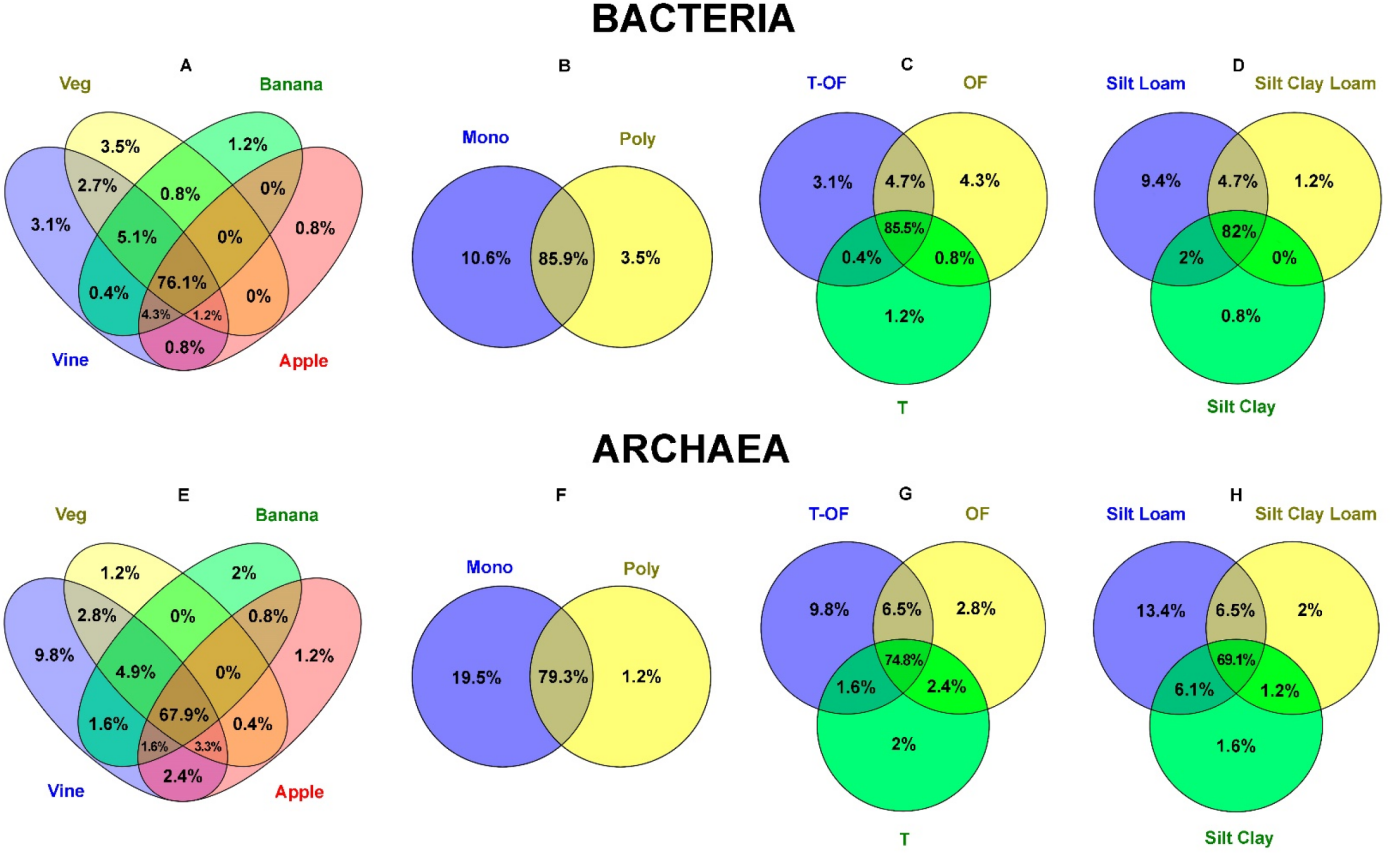
Venn diagram with the percentage of shared T‐RFs among different management practices and soil textures. Figures (A) to (D) are for bacterial communities. Figures (E) to (H) are for archaeal communities. (A) and (E) cultivated crops: vegetables (veg), banana, vine, apple; (B) and (F) cropping system: monoculture (mono) and polyculture (poly); (C) and (G) mode of production: transition to organic farming (T‐OF), organic farming (OF), traditional (T); (D) and (H) soil texture: silt loam, silt clay loam, silt clay.

Bacteria is the microbial group that showed a higher percentage of shared T‐RFs for the three analysed management practices and also for the soil texture (Figure 9A–D; Figure S1). This means that there was little influence of the management practices and soil texture on the presence of most of T‐RFs. However, it is worth noting that, there were 10.6% of total T‐RFs that only appeared in agrosystems under monoculture, and regarding soil texture, 9.4% were only found in silt loam soils.

The management practices and soil texture showed a higher influence on the archaeal community (Figure 9E–H; Figure S1). There are 9.8% of T‐RFs that were specific from vine agrosystems and agrosystems transitioning to organic farming, while 19.5% appeared only in monoculture and 13.4% only in silt loam agrosystems.

**FIGURE 10:**
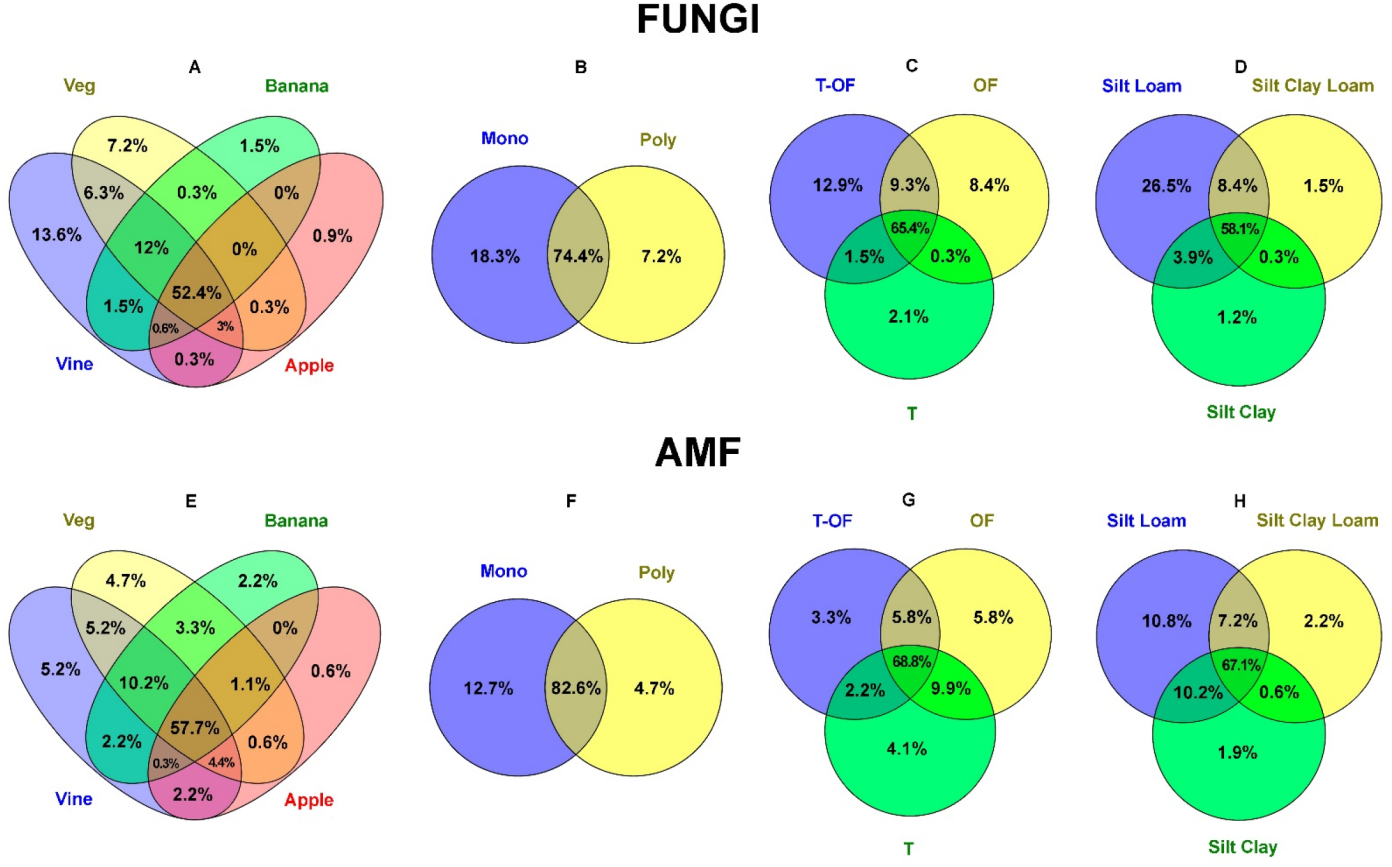
Venn diagram showing the percentage of shared T‐RFs among different management practices and soil textures. Figures (A) to (D) are for fungal communities. Figures (E) to (H) are for AMF communities. (A) and (E) cultivated crops: vegetables (veg), banana, vine, apple; (B) and (F) cropping system: monoculture (mono) and polyculture (poly); (C) and (G) mode of production: transition to organic farming (T‐OF), organic farming (OF), traditional (T); (D) and (H) soil texture: silt loam, silt clay loam, silt clay.

Although there was a core of fungal T‐RFs that were present in all management practices and soil textures, these also influenced the distribution of some T‐RFs (Figure 10A–D; Figure S2). Vine (13.6%) and vegetable (7.2%) agrosystems had more unique T‐RFs than banana (1.5%) and apple (0.9%) agrosystems. The apple tree agrosystem showed very few unique T‐RFs and the main core of the fungal community from this agrosystem was shared with the other five agrosystems. Monoculture (18.3%) and transitioning to OF (12.9%) agrosystems showed a higher percentage of unique T‐RFs, as well as the silt loam agrosystems (26.5%).

The AMF group followed a similar pattern as the general fungi (Figure 10E–H; Figure S2). However, AMF showed a higher percentage of shared T‐RFs among management practices and soil textures, and in this case, the agrosystems under organic farming were the ones with a higher percentage of unique T‐RFs (5.8%).

Several studies reported the effect of management practices and soil texture on microbial communities (Hamarashid et al., 2010; Flores et al., 2022; Zhang et al., 2019). In this study, more than 50% of the total T‐RFs were shared among management practices and soil textures, highlighting that there was a core of T‐RFs that are stable under different conditions. Bacteria was the microbial community with a higher percentage of shared T‐RFs. The effects of management practices, even if under long‐term application, can be influenced by the sampling time (Fernandez‐Gnecco et al., 2022; Megyes et al., 2021). Our data refer to samples collected at four different time points of the year, over 2 years, so it was not possible to find strong differences among management practices or soil texture, mainly for bacteria. But it led us to infer that there was a core of microbial taxa that might decrease or even be shifted over some time but tend to reappear. Nonetheless, there are management practices and soil textures that are more likely to have unique T‐RFs in the four microbial groups. These T‐RFs are linked to taxa that seem to grow in specific conditions.

## FINAL REMARKS

This study is the first report characterising soil microbial communities in agrosystems from Madeira Archipelago, a region with mixed Subtropical and Mediterranean climatic elements.

In summary, the structure of bacterial communities was revealed to be stable under different edaphoclimatic and management practices found in the agrosystems. The predominance of copiothrophic groups, suggests that the nutrient content of the soils was the driver of these communities. Concerning archaeal communities, unexpectedly, Euryarcheaota was the dominant phylum and we found that agrosystems from Madeira Archipelago provide niches for methanogens. However, further studies are required to infer their activity in field conditions. The composition and diversity of archaeal communities changed among sites. Beta‐diversity shows that communities from PS and QL stand out from the others. The management practices coupled with soil texture and possibly the annual accumulated precipitation determined the lower richness, but higher evenness and shared T‐RFs observed in these two agrosystems. Fungal communities did not change greatly in composition among agrosystems at the class level, but the diversity indices changed. In addition, the management practices and soil texture showed some influence, determining the presence of about 26%–47% of the total T‐RFs. On the other hand, the AMF fungal group seems to be more influenced by the agrosystems as a whole, than by only management practices or soil texture. The dissimilarity observed between agrosystems highlights the site specificity of the AMF communities' structure. However, there is a core of T‐RFs that is predominant in all sites.

Obtaining data from variations in microbial communities across different agrosystems provides a baseline for long‐term monitoring. This data also gives insights into the influence of climate change on microorganism composition and diversity. Such changes can have significant effects on productivity, resilience, and sustainability. It is intended that this monitoring be carried out for an extended period to provide more accurate information and contribute to the robustness of prediction models.

## AUTHOR CONTRIBUTIONS

**Maria Cristina O. Oliveira:** Conceptualization (equal); data curation (equal); formal analysis (equal); investigation (equal); methodology (equal); software (equal); validation (equal); visualization (equal); writing – original draft (equal); writing – review and editing (equal). **Carla Ragonezi:** Conceptualization (equal); data curation (equal); formal analysis (equal); investigation (equal); methodology (equal); software (equal); validation (equal); visualization (equal); writing – original draft (equal); writing – review and editing (equal). **Sofia Valente:** Conceptualization (equal); investigation (equal); methodology (equal); writing – review and editing (supporting). **José G. R. de Freitas:** Conceptualization (equal); investigation (equal); writing – review and editing (supporting). **Miguel Â. A. Pinheiro de Carvalho:** Funding acquisition (lead); project administration (lead); resources (equal); supervision (lead).

## CONFLICT OF INTEREST STATEMENT

The authors declare no conflict of interest.

## Supporting information


**Figure S1:** UpSet plot showing how many unique T‐RFs of bacteria and archaea, are shared between and among the management practices and soil texture.
**Figure S2:** UpSet plot showing how many unique T‐RFs of fungi and AMF are shared between and among the management practices and soil texture.

## Data Availability

The data that support the findings of this study are available from the corresponding author upon reasonable request.
