# Supplementary material for: Microorganism community structure: A characterisation of agrosystems from Madeira Archipelago
Source: Environ Microbiol Rep. 2024 Jan 24;16(1):e13227. doi: 10.1111/1758-2229.13227 (PMC10866076; doi:10.1111/1758-2229.13227)
Supplement: Supplementary file 1 — Figure S1: UpSet plot showing how many unique T‐RFs of bacteria and archaea, are shared between and among the management practices and soil texture. Figure S2: UpSet plot showing how many unique T‐RFs of fungi and AMF are shared between and among the management practices and soil texture. [file EMI4-16-e13227-s001.docx]

# Supplementary Materials


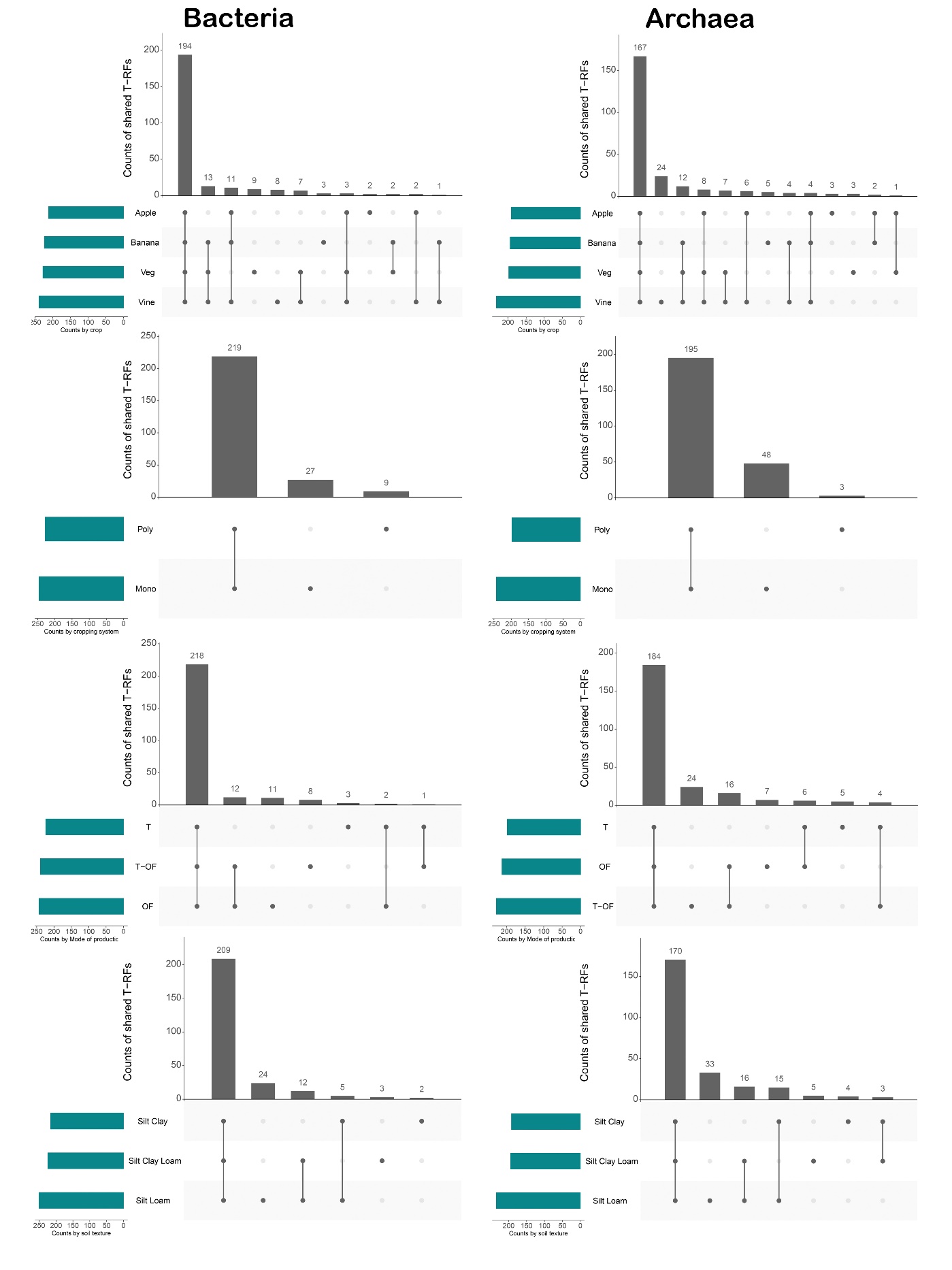


Fig. S1. UpSet plot showing how many unique T-RFs of bacteria and archaea, are shared between and among the management practices and soil texture.


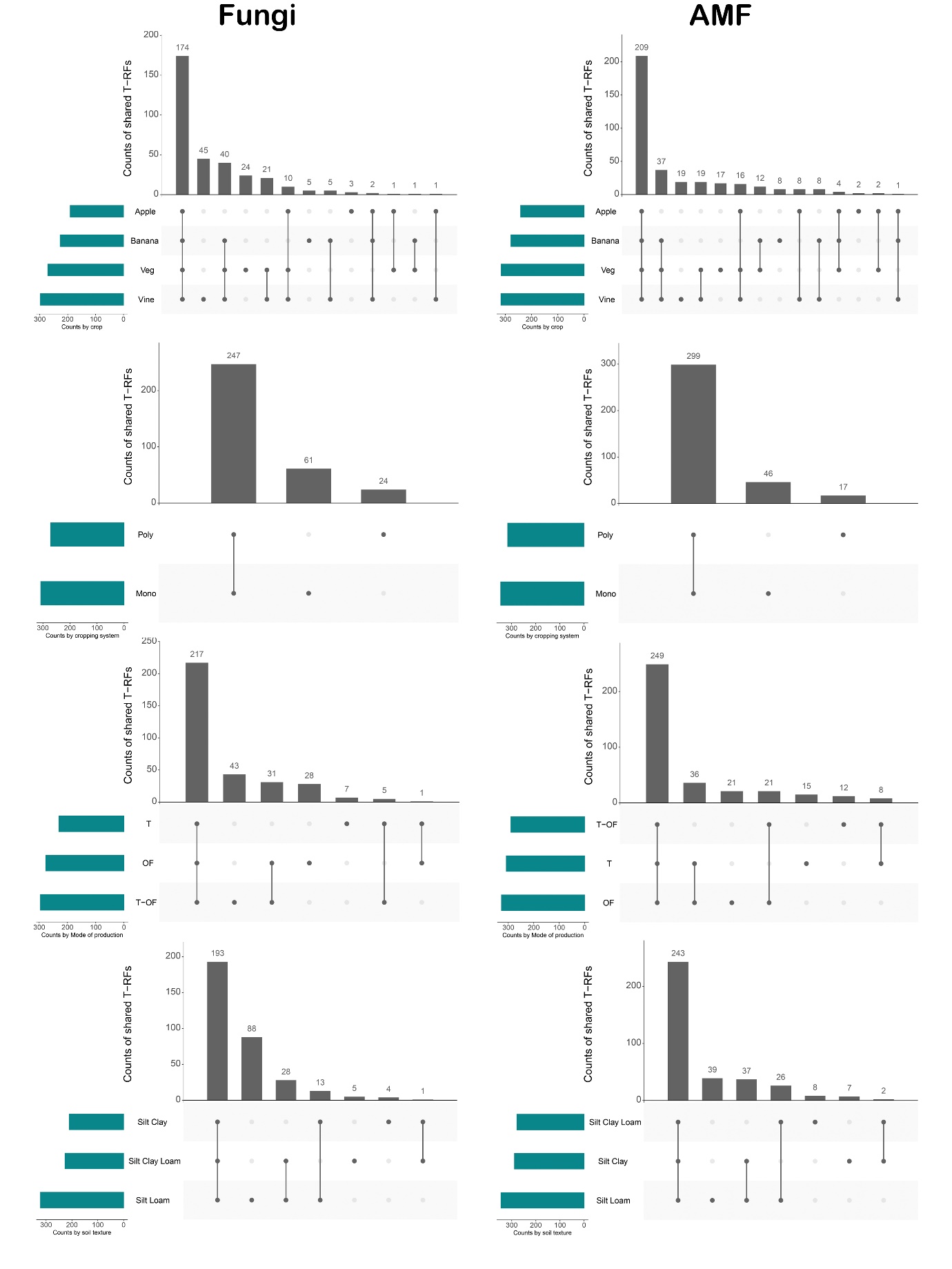


Figure S2. UpSet plot showing how many unique T-RFs of fungi and AMF are shared between and among the management practices and soil texture.
